# Learning Transversus Abdominis Activation in Older Adults with Chronic Low Back Pain Using an Ultrasound-Based Wearable: A Randomized Controlled Pilot Study

**DOI:** 10.3390/jfmk10010014

**Published:** 2025-01-01

**Authors:** Luis Perotti, Oskar Stamm, Hannah Strohm, Jürgen Jenne, Marc Fournelle, Nils Lahmann, Ursula Müller-Werdan

**Affiliations:** 1 Department of Geriatrics and Medical Gerontology, Charité—Universitätsmedizin Berlin, Corporate Member of Freie Universität Berlin and Humboldt-Universität zu Berlin, 13347 Berlin, Germany; 2 Fraunhofer Institute for Digital Medicine MEVIS, 28359 Bremen, Germany; 3 Department of Ultrasound, Fraunhofer Institute for Biomedical Engineering, 66280 Sulzbach, Germany

**Keywords:** chronic low back pain, biofeedback, segmental stabilization, ultrasound imaging, preferential activation ratio, real-time, deep learning

## Abstract

**Background/Objectives**: Chronic low back pain (CLBP) is prevalent among older adults and leads to significant functional limitations and reduced quality of life. Segmental stabilization exercises (SSEs) are commonly used to treat CLBP, but the selective activation of deep abdominal muscles during these exercises can be challenging for patients. To support muscle activation, physiotherapists use biofeedback methods such as palpation and ultrasound imaging. This randomized controlled pilot study aimed to compare the effectiveness of these two biofeedback techniques in older adults with CLBP. **Methods**: A total of 24 participants aged 65 years or older with CLBP were randomly assigned to one of two groups: one group performed self-palpation biofeedback, while the other group used real-time ultrasound imaging to visualize abdominal muscle activation. Muscle activation and thickness were continuously tracked using a semi-automated algorithm. The preferential activation ratio (PAR) was calculated to measure muscle activation, and statistical comparisons between groups were made using ANOVA. **Results**: Both groups achieved positive PAR values during all repetitions of the abdominal-draw-in maneuver (ADIM) and abdominal bracing (AB). Statistical analysis revealed no significant differences between the groups in terms of PAR during ADIM (*F*(2, 42) = 0.548, *p* = 0.58, partial *η*^2^ = 0.025) or AB (*F*(2, 36) = 0.812, *p* = 0.45, partial *η*^2^ = 0.043). Both groups reported high levels of exercise enjoyment and low task load. **Conclusions**: In conclusion, both palpation and ultrasound biofeedback appear to be effective for guiding older adults with CLBP during SSE. Larger studies are needed to confirm these results and examine the long-term effectiveness of these biofeedback methods.

## 1. Introduction

Chronic back pain is a widespread condition that leads to considerable restrictions in the quality of life of those affected, a high burden of disease, and a major economic impact [1,2,3]. The prevalence of low back pain in the older population is between 21% and 75% internationally [4]. The 12-month prevalence of chronic back pain in Germany is 28% for women and 17.4% for men above the age of 70 years [5]. In addition to the use of medication, physiotherapy plays an important role in the treatment of chronic low back pain (CLBP) [6,7]. Segmental stabilization exercises (SSEs) have been used for years to reduce pain as well as improve functional disability, and evidence in the area of pain management is well-documented in the literature [8,9,10,11]. SSEs aim to increase core stability through targeted training and by learning to selectively contract the deep abdominal and back muscles [8,12,13]. The abdominal draw-in maneuver (ADIM) and abdominal bracing (AB) are two prominent forms of exercise. While the ADIM involves contracting the transversus abdominis muscle (TrA) in isolation from the internal abdominal oblique muscle (OI) and external abdominal oblique muscle (OE), core stability training with the AB is achieved by contracting all three muscles and aiming to increase intra-abdominal pressure. Patients often struggle to learn how to perform the exercises correctly [14,15,16], which is why biofeedback is often used in practice to help with assessment and visualization [9,17]. In the context of SSE, the deep abdominal muscles are often palpated with the therapist’s or patient’s finger via the abdominal wall to feel whether the TrA contracts during the exercises [18]. Rehabilitative ultrasound imaging (RUSI) has also been used as biofeedback for several years and has been investigated as a potentially more precise biofeedback method [19,20,21]. RUSI provides real-time visual feedback for both the therapist and the patient on the state of contraction of all muscles involved in SSE. In order to quantify the accuracy of exercise execution during SSEs or to measure training progress, the thickness of the muscles during exercise can be measured using the ultrasound image. These measurements allow the preferential activation ratio (PAR) to be calculated. The PAR calculates the degree of isolated contraction of the TrA in relation to the OI and OE by comparing the thickness of the muscles during contraction and relaxation [22,23]. While both RUSI and palpation have been widely used in practice [24], there is still a gap in the understanding of which of the two methods is better suited as feedback for older adults with CLBP to achieve a positive PAR during ADIM and AB. Most studies measure the thickness of the muscles in the ultrasound image at precisely defined times during exercise performance (usually during complete exhalation) [19,23]. In addition, stationary wired ultrasound systems have predominantly been used for imaging. In this study, a semi-automated muscle detection, segmentation, and thickness measurement algorithm was used to continuously measure muscle thickness during SSEs. In addition, a mobile wireless ultrasound system was used with a specially developed holder prototype to allow greater mobility for the participants and ensure continuous positioning between exercises. It was investigated as to which biofeedback method (palpation vs. RUSI) was more suitable for patients with CLBP aged 65 years and older for learning the correct exercise performance for SSEs. The primary objective of this study is to assess whether exercise performance of SSEs differ between the palpation group and the RUSI group, as measured by the averaged preferential activation ratio of the deep abdominal muscles in older adults with chronic low back pain. As secondary outcomes, we will investigate the differences in the preferential activation ratio within both groups between the three exercise repetitions. We will also analyze the differences in perceived task load and enjoyment in SSE performance between the RUSI and the palpation group. As an exploratory objective, this study aims to investigate how participants of the RUSI group evaluate the user experience of the ultrasound system as a method for providing biofeedback on SSE performance.

## 2. Materials and Methods

### 2.1. Study Design

The purpose of this randomized controlled pilot study is to investigate two different feedback methods during SSEs, according to Richardson and Jull [13], in older adults with CLBP. In the first group, feedback on exercise execution was obtained through the manual palpation of the deep abdominal muscles (palpation group, PG) by a physiotherapist and by the participants themselves. The second group received feedback through a real-time ultrasound image of the deep abdominal muscles (rehabilitative ultrasound imaging group: RUSI group, RG). The study was approved by the Ethics Committee of the Charité—Universitätsmedizin Berlin (EA1/165/23). This study was registered to the German Clinical Trials Register (DRKS-ID: DRKS00032676; UTN: U1111-1298-8176). We followed the CONSORT guideline and added the checklist to the Supplementary Material [25].

### 2.2. Study Participants

Participants in this study were included based on specific criteria to ensure the relevance and safety of the intervention. Inclusion criteria required individuals to have non-specific CLBP persisting for longer than six months, be aged 65 years or older, and have the ability to move independently without help. Conversely, participants were excluded if they had a diagnosis of mild cognitive impairment or dementia, sensory or motor deficits, or were unable to actively perform the SSE. Additional exclusion criteria included fractures of the spine within the last six months, spinal malignancies or tumors, spondylitis, spondylodiscitis, fibromyalgia, a history of intervertebral disk surgery, the presence of pacemakers, venous thrombosis, or the use of muscle relaxants. Additional characteristics of the sample, such as physical activity levels, underlying medical conditions, and other relevant factors, were collected as part of the baseline data and are presented in Table 1.

### 2.3. Study Procedure and Assessments

**Figure 1:**
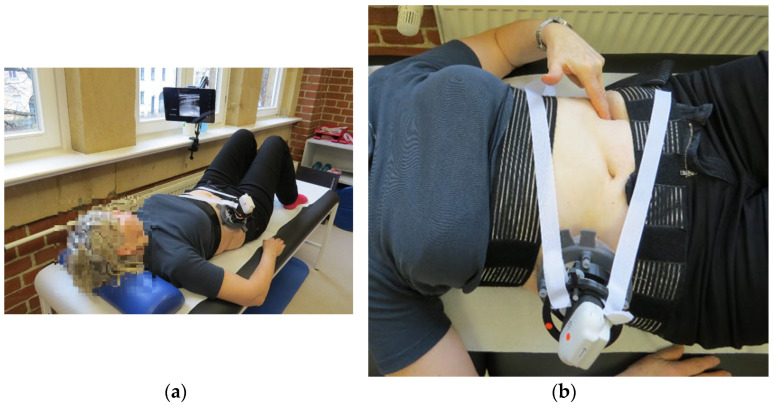
(**a**) Study setting for RG; (**b**) study setting for the PG.

**Figure 2:**
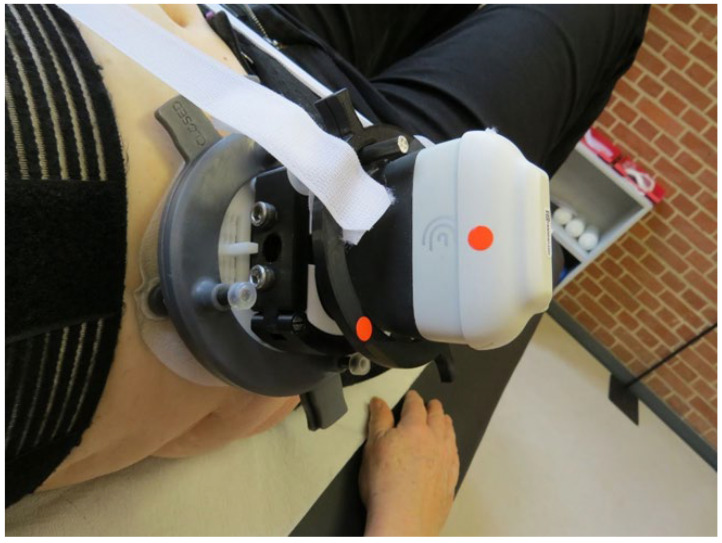
Custom-built holder for the Clarius L15 HD3.

A person not involved in the study randomly assigned each participant to one of the two groups. Group affiliation was handed over to the study staff in sealed envelopes before the respective study appointments. Potential participants were informed about the study and were able to ask questions. The setup of the system used in the study (ultrasound system with holder, Figure 1 and Figure 2) and the procedure for the various physiotherapeutic exercises were explained. After signing the informed consent form, the envelope with the group assignment and the subject ID was opened and the participants were asked to complete the general data questionnaire (age, sex, marital status, highest education, physical activities, etc.). The Affinity for Technology Interaction Scale—Short Version (ATI-S) [26] was used to determine the participants’ affinity for technology. The participants’ health status concerning their CLBP was assessed using the Numerical Rating Scale (NRS) [27] for their current condition, and the Roland Morris Disability Questionnaire (RMDQ) [28] was utilized for assessing their functional limitations. The Chronic Pain Grade Questionnaire (CPGQ) [29] was used to classify chronic pain, and the PEG [30] was used as an additional screening tool for pain intensity and interference. All participants were introduced to the anatomical structure of the deep abdominal muscles. The ultrasound system was then placed on the subject’s body with the holder and the SSEs were performed. Finally, the participants completed the National Aeronautics and Space Administration Task Load Index (NASA TLX) [31] with weighted ratings for measuring subjective workload during exercise performance. The short version of the Physical Activity Enjoyment Scale (PACES-S) [32] was used to measure the enjoyment of physical activity. The User Experience Questionnaire (UEQ) [33] was used in the RG for evaluating the experience of using the ultrasound system as a biofeedback tool during SSE execution. All assessments and questionnaires were in the German language.

### 2.4. Ultrasound System

The ultrasound system used was the Clarius L15 HD3 with a high-frequency linear ultrasound transducer (Clarius Mobile Health, Vancouver, BC, Canada), which has a maximum penetration depth of 7 cm and a frequency of between 5 and 15 MHz. The system features 192 piezo elements and 8 beamformers, a pitch of 260 μm, and a field of view of 50 mm. The recordings were made in B-mode, and the penetration depth, TGC, and other settings were set individually for each participant.

### 2.5. Positioning of the Ultrasound Transducer

The transducer was positioned horizontally with a slight rotation in a caudal direction between the lowest rib and the anterior iliac crest. The transducer was positioned approximately at the level of the subject’s umbilicus and the fascia semilunaris had to be visible 1 to 2 cm from the edge of the ultrasound image [23]. Similarly, the TrA, OI, and OE muscles and their movements during contraction had to be clearly depicted in the ultrasound image (Figure 1). Image settings such as penetration depth and contrast were set individually. The system was connected to an iPad to display the ultrasound image. We used a prototype of a specifically designed holder (Figure 2) to ensure stable positioning. The design copyright for the holder is held by DITABIS AG—Digital Biomedical Imaging Systems AG. In the RG, the iPad was positioned so that it was clearly visible to the participants and the study personnel during the exercises; in the PG, only the study personnel could see the screen.

### 2.6. Exercise Concept

The exercises ADIM and AB were chosen as both interventions demonstrated efficacy in activating the TrA and enhancing core stability. Prior studies highlighted their suitability for improving neuromuscular control and functional outcomes, particularly in patients with low back pain [34,35]. Two exercises were performed in a supine position and four exercises in a standing position. Both ADIM and AB were performed while lying down, with three repetitions each. The exercises were performed three times in the supine position to overcome the participants’ lack of previous experience and to make it easier for them to learn the two methods. In addition, the feedback given after each repetition was intended to facilitate efficient learning. By repeating the exercises several times, it was possible to follow the learning process retrospectively. While standing, ADIM was performed in the following positions: flexed weight-bearing posture, fully extended anti-gravity posture, half squat with elevated arms, closed-chain lunge with weights, and flexed weight-bearing posture on an unstable surface. The exercise selection in the standing position is based on Richardson and Hides’ proposed training method of segmental control in the closed movement chain [11]. The SSEs in a standing position were performed twice each, due to time resources and the faster adaptation observed compared to the basic exercise in the supine position made additional repetitions unnecessary. The participants in both groups received identical verbal instructions while performing the exercises. We utilized two forms of augmented feedback timing within both groups. Verbal feedback was always terminal feedback, aligning with the knowledge of results. Furthermore, the RG was provided with real-time ultrasound images concurrently, representing the knowledge of performance. Similarly, the PG received concurrent tactile or somatosensory feedback, thus also acquiring knowledge of performance.

### 2.7. Muscle Tracking Algorithm

**Figure 3:**
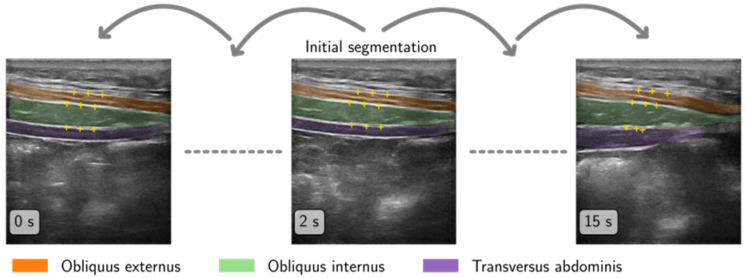
Muscle detection and segmentation from the initial frame, participant ID 07 (male, 70 years old). The yellow markings indicate measuring points for the thickness measurements.

**Figure 4:**
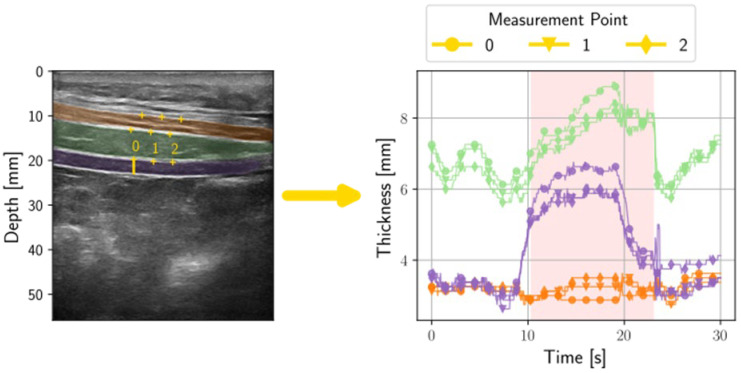
Measurement of muscle thickness during exercise using three measurement points, participant ID 07 (male, 70 years old). Three graphs are displayed for each muscle (orange: OE, green: OI, purple: TrA), each representing the thickness of the muscle at one of three measurement points. The different shapes of the points on the graph indicate which measurement point (0, 1, 2) is shown. The red range in the graph indicates the automated detection of the contraction phase of the muscles.

Muscle thickness was assessed in a semi-automatic way by first segmenting one initial frame (Figure 3). As the initial frame, the one with the highest brightness after filtering with a Sobel edge kernel was determined with the idea to retrieve a frame where the muscle boundaries are visible best. A Viterbi-based algorithm [36] then determined the muscle fascia by identifying the brightest paths through the selected frame. The resulting segmentation was presented to the study staff, who corrected the segmentation in case it was not satisfactory. The initial segmentation was then propagated using an optical flow tracking algorithm to retrieve a segmentation for every frame of the video. Additionally, three measurement points per muscle were tracked and were placed in the middle of the upper fascia of each muscle in the initial segmentation (Figure 4). Tracking was performed using the Lucas–Kanade method [37,38] with initial parameters of Sigma = 0.04, fgs lambda = 5000, and fgs sigma = 3. The reference frame for tracking was determined by selecting the one with the highest correlation from the set of already segmented frames, which is not necessarily the direct predecessor of the current frame. The initial tracking parametrization was sufficient for 89 of the 144 videos (62%). For the remaining videos, tracking parameters were tuned manually to obtain a sufficient result. In 25 videos, an additional frame was segmented to help stabilize the tracking.

### 2.8. Statistical Analysis

In order to investigate the extent to which the learning of the SSEs differed between the groups, the PAR was calculated from the measurements for the individual muscle thicknesses between the relaxation and contraction phases for each exercise repetition. A mean for all three measurement points seen in Figure 4 was calculated. As the image quality during the exercises in standing position was not sufficient for muscle thickness measurement, the calculations were only carried out for the exercises in the supine position. A repeated measurement ANOVA was performed separately for ADIM and AB. The prerequisites for ANOVA performance were tested. A test for normal distribution was also carried out to analyze the change within the two groups in relation to the PAR and for the comparisons of the secondary assessments between the groups. As a normal distribution was present, t-tests for repeated measurements were carried out. Where normal distribution was not present, parametric tests were used. An alpha level of 0.05 was used for the interpretation of the results. Data analysis was performed using SPSS (version 29; IBM SPSS, Chicago, IL, USA).

## 3. Results

**Figure 5:**
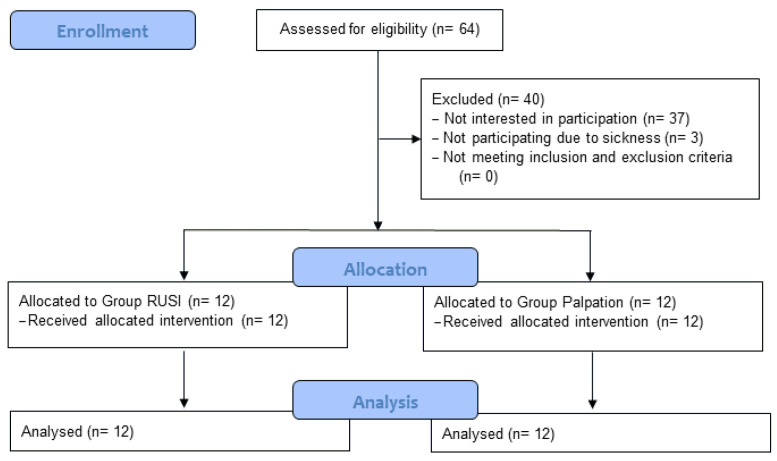
CONSORT flow diagram.

Participants were recruited and the study was carried out between October and December 2023. A total of 24 participants (12 per group) took part in the study (Figure 5, Table 1). The mean age of the participants was 76.08 years (*SD* = 3.90) in the RG and 77.00 years (*SD* = 4.69) in the PG. The proportion of female participants in both groups was 75%. No significant differences in demographic data, in affinity for technology, or in the assessments for chronic pain characterization were evident.

### 3.1. Muscle Thickness During SSE

Table 2 shows the mean muscle thickness for the three repetitions individually and the means for all repetitions. The table shows the muscle thicknesses for the two exercises, ADIM and AB, in the supine position at rest and contracted. Using the continuous tracking and measurements, we calculated the PAR, shown in Table 3. Out of a total of 144 videos, 5 videos of patients in the supine position were excluded from the analysis due to poor image quality; 4 recordings for AB (3 of Repetition 2 and 1 of Repetition 3) and 1 of the ADIM (Repetition 1) were not included.

### 3.2. Comparison of PAR Between Groups

#### 3.2.1. Abdominal Drawing-In Maneuver

The means and standard deviations for the PAR during ADIM are presented in Table 3. A repeated-measures ANOVA was performed to evaluate the effect of group assignment on PAR during ADIM. Mauchly’s test indicated that the assumption of sphericity had been met, χ^2^ (2) = 5.66, *p* = 0.06. The effect of group assignment on PAR during ADIM was not significant (*F*(2, 42) = 0.548, *p* = 0.58, partial *η*^2^ = 0.025).

#### 3.2.2. Abdominal Bracing

A repeated-measures ANOVA was performed to analyze the effect of group assignment on PAR during AB. The means and standard deviations for PAR during AB are presented in Table 3. Mauchly’s test indicated that the assumption of sphericity had been met, at χ^2^ (2) = 0.23, *p* = 0.89. The effect of group assignment on PAR during AB was also not significant, at F(2, 36) = 0.812, *p* = 0.45, and partial *η*^2^ = 0.043.

### 3.3. Differences in PAR Between Exercise Repetitions During ADIM and AB

The results of paired t-tests analyzing the differences in the PAR measurements within the RG and PG indicate that there were no significant differences between any of the three exercise repetitions nor during ADIM or AB (Table 4).

### 3.4. Task Load

**Figure 6:**
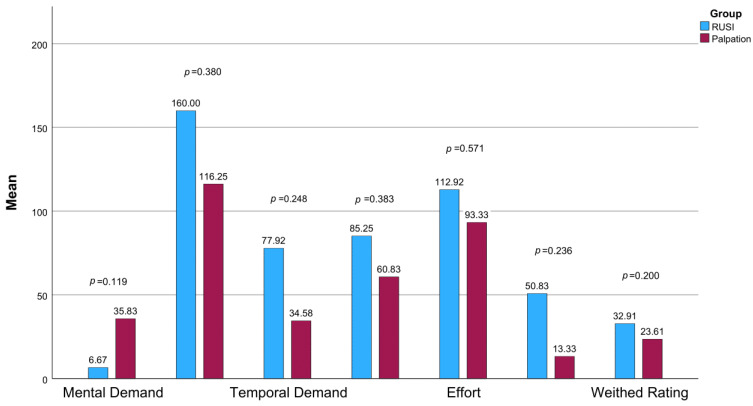
Comparison of the results from the NASA TLX subscales between the RG and PG.

When looking at the results of the adjusted NASA TLX of the RUSI and the PG, the highest score (i.e., the highest subjective burden/workload) was reached in the physical demand subscale (Figure 6). The RG mental demand was perceived as lowest (M = 6.67, *SD* = 8.07), while the PG reported the lowest workload in the frustration subscale (M = 13.33, *SD* = 32.00). When comparing the values achieved for the subscale between the two groups, the *t*-test for independent samples revealed no significant differences in any of the subscales.

### 3.5. PACES-S

When evaluating the enjoyment that the participants had while performing the exercises, the participants in the RG achieved a median overall PACES-S score of 18.00 (IQR = 5). The people in the PG achieved a median of 15.00 (IQR = 6). The difference in scores between the two groups was not significant (U = 56.50, *p* = 0.38).

### 3.6. User Experience Questionnaire

**Figure 7:**
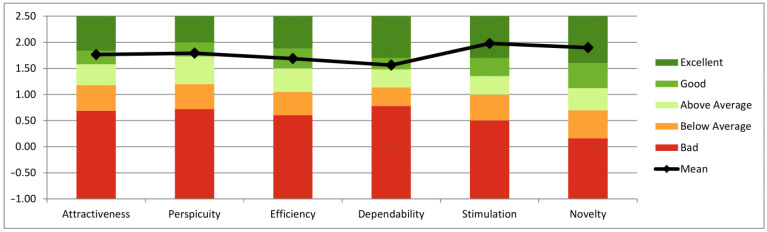
Interpretation of the UEQ scales for the RG as compared to the benchmark.

The ultrasound system as a biofeedback tool received scores indicating a positive evaluation of the user experience by the RG on all scales (Table 5). The highest scores were reached on the scales of stimulation (*M* = 1.98, *SD* = 0.63) and novelty (*M* = 1.90, *SD* = 0.87). Pragmatic quality was rated as having a score of 1.76, and hedonic quality was rated as having a score of 1.94. Compared to the benchmark, the scales of attractiveness, perspicuity, efficiency, and dependability were rated as good, and the scales of stimulation and novelty were rated as excellent (Figure 7).

## 4. Discussion

The study presented here includes an innovative methodology in the use of a mobile ultrasound system with a specially developed holder and continuous and semi-automated thickness measurements of the muscles. Positive PAR values were achieved for the ADIM across all repetitions and in both groups in the supine position. However, no significant difference was found between the groups and the individual repetitions.

Comparisons of the two feedback methods, RUSI and palpation, have already been published by Valentín-Mazarracin et al. [24], but they used RUSI only as a tool to assess the activation of the deep abdominal muscles not as biofeedback as a learning tool for patients. Valera-Calero et al. [39], on the other hand, compared RUSI as tactile or verbal biofeedback and found that it was more suitable for increasing muscle thickness. Lin et al. [40] also investigated RUSI as biofeedback in relation to its influence on preferential activation of the TrA, with RUSI proving to be effective as feedback for ADIM. Henry and Westervelt [41] also compared verbal feedback, palpations, and RUSI as forms of feedback for participants. They investigated the extent to which participants were able to perform three successful repetitions of abdominal hollowing during an initial session. RUSI proved to be the most successful feedback method. However, they did not quantify successful exercise execution by measuring muscle thickness and calculating PAR. In our study, no significant differences in PAR were found between the groups when comparing RUSI and palpation as biofeedback. Nevertheless, the exercises were predominantly performed correctly in both groups (as can be seen from the PAR values, Table 3). We also compared the PAR between the exercise repetitions within each group and found no significant differences (Table 4). Descriptively, however, it can be noted that the PAR values in both groups show a mostly positive trend towards higher PAR values with each repetition. The absence of statistical difference can either be attributed to the small sample size or the good overall exercise execution. Since the mean PAR was always positive, even in the first repetition, it can be assumed that the participants understood the goal of the ADIM right after the introduction and thus no differences were found between the repetitions. In future studies, longitudinal observations might be of interest.

The overall results of our study indicate that the biofeedback using ultrasound imaging is not inferior to traditional feedback via palpation. Since palpation feedback is usually provided by therapists, these findings might be crucial for future applications of the technology in non-clinical settings, such as home use without the supervision of a physiotherapist. As this was a pilot study designed to provide exploratory insights into the comparison of these two feedback methods, the results highlight promising opportunities for future applications for self-administered training.

Although there were no significant differences in subjective workload, as measured by the NASA TLX in any of the subscales, it can be observed that scores for the physical demand were highest in both groups and descriptively higher in almost all subscales for the RG than in the PG (Figure 6). In the requirements analysis in this research project, older adults with CLBP were asked about their attitudes towards an AI-supported system using RUSI for SSE. The participants expressed the concern that it might be difficult to simultaneously concentrate on an ultrasound image and the SSE execution [42]. However, considering the low scores for the mental demand subscale of the NASA TLX in the RG, this does not seem to have been an issue in the current study. No significant difference in mental demand between the two groups was evident. The enjoyment of the SSEs was high in both groups as measured by the PACES-S. There is no research in the current literature on the extent to which different forms of biofeedback influence enjoyment during SSEs. As there was no significant difference between the groups in our study, either the sample size is too small or the form of feedback has no influence on enjoyment. However, on a positive note, the RUSI group also demonstrated high PACES-S scores, which makes the use of this technology and feedback via ultrasound imaging seem viable in the target group of older people. The user experience in using RUSI with the ultrasound system also achieved a good to excellent rating in the UEQ (Figure 7). This reinforces the view that RUSI is suitable for learning and teaching SSEs for older adults with CLBP. Participants enjoyed the technology, which may be attributed to the novelty effect and their interest in ultrasound imaging, as reflected in their high affinity for technology and positive feedback on the innovative approach.

In our analysis, only measurements of muscle thickness in the supine position could be analyzed, as image quality and stability in the standing position were too limited for automated detection. Future analyses with extended image processing algorithms are conceivable here. Moghadam et al. [43] observed differences in PAR during ADIM and AB in regard to the postures in which SSEs were performed. This could not be confirmed in our study due to technical limitations. The participants in our study only had one session in which the SSEs were learned and muscle thicknesses were measured. No statement can be made about the sustainability of learning between the two groups.

### Limitations

The conducted study is highly innovative, particularly due to the partially automated detection of the muscles in the ultrasound image and the continuous measurements of muscle thickness during exercise performance. However, some limiting factors need to be considered when interpreting the results. Due to the pilot nature of the study, only a relatively small sample size could be included in the analysis, which is also reflected in the statistical significance of the results. A study with a larger number of participants should be conducted in the future. Subgroup analysis or controlling for factors such as age distribution, underlying condition, and prior experience with physical activity was not feasible with the small sample size. Likewise, no stratification (e.g., by gender) could be applied during randomization. As the system used was quite prone to movement due to the experimental mount location, palpitations in the PG could not be registered on the body side on which the ultrasound system was placed. Finally, it should be mentioned that the semi-automated muscle detection and thickness measurement introduced a certain inaccuracy of the measurements, which had to be manually adjusted by the study personnel.

## 5. Conclusions

By comparing real-time biofeedback delivered via RUSI to traditional palpation feedback, we provided initial evidence that both methods are equally effective for teaching and supporting the abdominal draw-in maneuver in older adults with chronic back pain. Participants in both groups demonstrated consistent improvements in their ability to perform ADIM across all repetitions, with no significant differences observed between groups or individual repetitions. The results highlight the potential of RUSI as a valuable tool for teaching, assessing, and enhancing exercise performance, particularly in the activation of deep abdominal muscles. The lack of significant differences between the two feedback methods suggests that ultrasound-based biofeedback is not inferior to traditional palpation. This insight may have significant implications for the future use of RUSI in non-clinical settings. Although no significant differences in subjective workload were found between the groups, participants reported high levels of enjoyment and positive user experiences with the ultrasound-based system. The good user experience and results in using RUSI for exercise may implicate its feasibility for settings like home environments, where supervision by a physiotherapist may not be possible. As this was a pilot study, these exploratory results provide a foundation for further research and development, offering promising perspectives for the broader application of ultrasound biofeedback technology in rehabilitation and beyond. 

## Figures and Tables

**Table 1:** Demographic characteristics of the study participants.

	RUSI	Palpation	*p*-Value
Number of Participants [*n*]	12	12	
Sex (male/female) [*n*]	3/9	3/9	1.00 ^a^
Age (*M* (*SD*)) [years]	76.08 (3.90)	77.00 (4.69)	0.61 ^b^
Age Range (min.–max.) [years]	70–82	70–85	
Highest Education Level [*n*]			0.63 ^c^
None	1	0	
Secondary School	0	1	
Intermediate School	3	1	
Grammar School	1	0	
University of Applied Sciences	1	2	
University	6	8	
Marital Status [*n*]			0.51 ^c^
Single	2	4	
Married	5	4	
Divorced	3	2	
Widowed	2	2	
BMI	26.16 (2.59)	26.44 (5.63)	0.88 ^b^
Moderate exercise activity (*M* (*SD*)) [days per week]	5.50 (1.78)	5.50 (1.88)	0.63 ^c^
Intense exercise activity (*M* (*SD*)) [days per week]	1.75 (1.42)	2.83 (1.95)	0.11 ^c^
ATI-S score (*M* (*SD*))	3.60 (1.32)	3.96 (1.32)	0.52 ^b^
CPGQ Pain Grade Score (*Mdn* (*IQR*))	1.00 (1)	1.50 (1)	0.65 ^c^
PEG Score (*M* (*SD*))	3.89 (2.03)	3.53 (2.13)	0.87 ^b^
NRS, Momentary Pain Assessment (*M* (*SD*))	2.67 (2.46)	3.08 (2.23)	0.56 ^c^
RMDQ Score (*M* (*SD*))	8.25 (3.96)	7.75 (4.94)	0.79 ^b^

Note: RUSI = rehabilitative ultrasound imaging; BMI = body mass index; ATI-S = Affinity For Technology Interaction Scale—Short Version; CPGQ = Chronic Pain Grade Questionnaire; NRS = Numeric Rating Scale; RMDQ = Roland Morris Disability Questionnaire. *p* ≤ 0.05. ^a^ Fischer’s exact test. ^b^ unpaired *t*-test. ^c^ Mann–Whitney U test.

**Table 2:** Muscle thicknesses at rest and during contraction during ADIM and AB.

Exercise	Muscle	Repetition 1		Repetition 2		Repetition 3		Repetition 1 to 3	
		Muscle Thickness [*M* (*SD*) in mm]	Muscle Thickness 95%-*CI* [in mm]	Muscle Thickness [*M* (*SD*) in mm]	Muscle Thickness 95%-*CI* [in mm]	Muscle Thickness [*M* (*SD*) in mm]	Muscle Thickness 95%-*CI* [in mm]	Muscle Thickness [*M* (*SD*) in mm]	Muscle Thickness 95%-*CI* [in mm]
ADIM	OE_R_	5.29 (2.45)	4.25, 6.32	5.13 (2.10)	4.25, 6.02	5.23 (2.42)	4.19, 6.28	5.22 (2.29)	4.67, 5.76
	OI_R_	9.51 (4.16)	7.76, 11.27	9.48 (4.38)	7.63, 11.33	9.03 (3.64)	7.45, 10.6	9.34 (4.02)	8.39, 10.31
	TrA_R_	4.79 (1.86)	4.00, 5.58	4.58 (1.76)	3.84, 5.32	4.42 (1.48)	3.78, 5.06	4.61 (1.69)	4.21, 5.00
	OE_C_	5.67 (3.06)	4.38, 6.96	5.33 (2.35)	4.34, 6.32	5.19 (2.33)	4.19, 6.20	5.4 (2.57)	4.79, 6.01
	OI_C_	10.63 (4.58)	8.69, 12.56	10.71 (4.78)	8.68. 12.72	10.36 (4.65)	8.35, 12.37	10.57 (4.61)	9.48, 11.66
	TrA_C_	5.45 (2.24)	4.50, 6.39	5.25 (1.50)	4.62, 5.89	5.65 (2.39)	4.61, 6.68	5.44 (2.05)	4.96, 5.93
AB	OE_R_	4.93 (1.96)	4.01, 5.75	5.12 (1.91)	4.25, 5.98	4.93 (1.79)	4.14, 5.73	4.99 (1.86)	4.54, 5.44
	OI_R_	9.24 (4.17)	7.48, 11.00	9.90 (5.09)	7.58, 12.21	9.13 (3.75)	7.46, 10.79	9.41 (4.30)	8.36, 10.46
	TrA_R_	4.40 (1.69)	3.68, 5.10	4.58 (1.98)	3.68, 5.48	4.47 (2.14)	3.53, 4.24	4.48 (1.91)	4.01, 4.94
	OE_C_	4.39 (1.47)	3.77, 5.01	4.55 (1.41)	3.91, 5.18	4.16 (1.41)	3.56, 4.77	4.36 (1.41)	4.02, 4.70
	OI_C_	8.61 (3.79)	6.99, 10.21	9.24 (4.33)	7.26, 11.21	8.64 (3.57)	7.09, 10.18	8.81 (3.85)	7.88, 9.74
	TrA_C_	4.32 (1.62)	3.64, 5.00	4.60 (2.09)	3.65, 5.55	4.81 (2.32)	3.81, 5.81	4.57 (2.00)	4.09, 5.06

Note: ADIM = abdominal draw-in maneuver; AB = abdominal bracing; OE_R_ = external abdominal oblique muscle at rest; OE_C_ = external abdominal oblique muscle contracted; OI_R_ = internal abdominal oblique muscle at rest; OIc = internal abdominal oblique muscle contracted; TrA_R_ = transversus abdominis muscle at rest; TrAc = transversus abdominis muscle contracted.

**Table 3:** PAR measurements during ADIM and AB.

Exercise	Group	Repetition 1		Repetition 2		Repetition 3		Repetition 1 to 3	
		PAR [*M*, (*SD*)]	PAR 95%-*CI* [LB, UB]	PAR [*M*, (*SD*)]	PAR 95%-*CI* [LB, UB]	PAR [*M*, (*SD*)]	PAR 95%-*CI* [LB, UB]	PAR [*M*, (*SD*)]	PAR 95%-*CI* [LB, UB]
ADIM	All participants	0.017 (0.040)	−0.000, 0.034	0.022 (0.040)	0.005, 0.039	0.035 (0.042)	0.017, 0.053	0.025 (0.031)	0.011, 0.038
	RUSI	0.029 (0.043)	0.001, 0.056	0.026 (0.033)	0.005, 0.047	0.048 (0.041)	0.021, 0.074	0.034 (0.031)	0.014, 0.054
	Palpation	0.004 (0.033)	−0.018, 0.026	0.018 (0.047)	0.014, 0.050	0.021 (0.041)	0.021, 0.074	0.014 (0.029)	−0.005, 0.034
AB	All participants	0.025 (0.047)	0.003, 0.047	0.016 (0.034)	0.000, 0.032	0.029 (0.063)	0.000, 0.059	0.023 (0.038)	0.006, 0.041
	RUSI	0.024 (0.028)	0.004, 0.044	0.022 (0.025)	0.004, 0.040	0.019 (0.037)	−0.007, 0.046	0.022 (0.025)	0.004, 0.040
	Palpation	0.025 (0.062)	−0.020, 0.070	0.020 (0.042)	−0.020, 0.040	0.040 (0.083)	−0.020, 0.099	0.025 (0.049)	−0.010, 0.060

Note: PAR = preferential activation ratio; LB = lower bound; UB = upper bound; ADIM = abdominal draw-in maneuver AB = abdominal bracing; RUSI = rehabilitative ultrasound Imaging.

**Table 4:** Comparison of PAR measurements during ADIM and AB between repetitions.

Exercise	Group	Repetition 1 and 2			Repetition 2 and 3			Repetition 1 and 3		
		*t*-Value	*p*-Value	*d*	*t*-Value	*p*-Value	*d*	*t*-Value	*p*-Value	*d*
ADIM	RUSI	0.92	0.85	0.05	−1.165	0.13	0.44	−1.942	0.08	0.52
	Palpation	−0.627	0.54	0.18	−0.176	0.86	0.05	−1.178	0.11	0.54
AB	RUSI	0.516	0.62	0.16	−1.358	0.21	0.43	−0.439	0.67	0.13
	Palpation	0.221	0.83	0.07	0.267	0.80	0.08	0.178	0.97	0.01

Note: ADIM = abdominal draw-in maneuver; AB = abdominal bracing; RUSI = rehabilitative ultrasound imaging.

**Table 5:** Results of the UEQ scales for the RG.

UEQ Scales	*M*	*SD*
Attractiveness	1.76	0.73
Perspicuity	1.79	0.83
Efficiency	1.69	0.60
Dependability	1.56	0.70
Stimulation	1.98	0.63
Novelty	1.90	0.87

Note: UEQ = User Experience Questionnaire.

## Data Availability

The data analyzed in this study are available on request from the corresponding author.

## Supplementary Materials

The following supporting information can be downloaded at: https://www.mdpi.com/article/10.3390/jfmk10010014/s1.

## Abbreviations

AB	Abdominal Bracing
ADIM	Abdominal Draw-In Maneuver
CLBP	Chronic Low Back Pain
CPGQ	Chronic Pain Grade Questionnaire
NASA TLX	National Aeronautics and Space Administration Task Load Index
NRS	Numeric Rating Scale
OI	Internal Abdominal Oblique Muscle
OE	External Abdominal Oblique Muscle
PACES-S	Physical Activity Enjoyment Scale-Short
PAR	Preferential Activation Ratio
PG	Palpation Group
RG	RUSI Group
RMDQ	Roland Morris Disability Questionnaire
RUSI	Rehabilitative Ultrasound Imaging
SSE	Segmental Stabilization Exercise
TrA	Transversus Abdominal Muscle
UEQ	User Experience Questionnaire
